# The Role of Moringa Leaf Extract as a Plant Biostimulant in Improving the Quality of Agricultural Products

**DOI:** 10.3390/plants11172186

**Published:** 2022-08-23

**Authors:** Nita Yuniati, Kusumiyati Kusumiyati, Syariful Mubarok, Bambang Nurhadi

**Affiliations:** 1 Faculty of Agriculture, Universitas Padjadjaran, Jalan Raya Bandung-Sumedang Kilometer 21 Jatinangor, Sumedang 45363, Indonesia; 2 Faculty of Agro-Industrial Technology, Universitas Padjadjaran, Jalan Raya Bandung-Sumedang Kilometer 21 Jatinangor, Sumedang 45363, Indonesia

**Keywords:** moringa leaf extract, biostimulant, quality, bioactive compound, nutrient

## Abstract

Ensuring high-quality agricultural products has become important in agriculture since society’s standard of living has risen. Meanwhile, *Moringa oleifera* L. leaf extract (MLE) has been used as a plant biostimulant to improve product quality. The effectiveness of MLE is associated with its beneficial components, consisting of nutrients, phytohormones, secondary metabolites, amino acids, and bioactive compounds. Previous studies have been carried out to find the effects of MLE application on the quality of different crops, including basil, kale, spinach, maize, radish, brinjal, pepper, tomato, grape, strawberry, and more. The results are generally positive concerning physical, nutritional, and chemical qualities. This review comprises recent findings regarding MLE application as a plant biostimulant to increase quality attributes, with its underlying mechanism.

## 1. Introduction

During the last decade, plant quality traits have become increasingly noticeable in agriculture, with society’s standard of living rising [1]. Agricultural products generally offer better shapes, sizes, levels of firmness, colors, and sensory characteristics (e.g., flavors and tastes) [2]. The health benefits, bioactive compounds, and nutritional qualities of agricultural products are also consumer concerns [3,4,5]. Consequently, the demand for high-quality products is increasing [6] and quality approaches have become important subjects of various studies.

The use of plant biostimulants is one of the strategies utilized to achieve high-quality products in a sustainable manner. Biostimulants are the products derived from biological materials to improve plant productivity, including yield, quality, or production efficiency, due to the presence of plant growth regulators, essential nutrients, and plant protective compounds [7]. These constituents alter the metabolism, signaling, and hormonal regulation of plants during growth and development [8]. Previous studies have reported the positive effects of biostimulants on product qualities under normal and stressful conditions [9,10]. Biostimulants, due to their high antioxidant capacities, are used to neutralize oxidative stress, preventing a drop in the yield and quality [11,12]. 

The various parts of higher plants, e.g., seeds, roots, or leaf extracts, can be used as biostimulant feedstock [13]. Research shows that *Moringa oleifera* L. leaf extract (MLE) is a potential biostimulant that could improve product quality [14]. It belongs to the Moringaceae family and is native to India [15]. Moreover, moringa is a fast-growing plant cultivated in the tropics and sub-tropic areas [16]. It produces plenty of biomass and its leaf contains numerous nutrients, vitamins, phytohormones, and secondary metabolites [17,18]. MLE obtained from a fresh leaf also comprises high antioxidants and osmoprotectants, including proline, amino acid, soluble sugar, α-tocopherol, and glutathione [19,20].

Previous studies revealed that MLE applications were highly recommended to increase the physical and chemical quality attributes of plants [21,22,23,24]. As MLE is an effective and sustainable alternative to improve quality, research efforts have been undertaken to understand its mechanism and how it yields positive quality outcomes. Recent review papers on MLE have focused on growth, yield, and abiotic stress tolerances, while research is limited concerning the quality. Therefore, this review provides information about the effects of MLE on several quality traits of agricultural products and its underlying mechanism.

## 2. Phytochemical Compositions and Application of MLE into Plants

MLE comprises various phytochemical compounds and its content depends on the extraction process. A common method for extracting moringa leaf for biostimulant purposes involves using a fabricated local machine or home blender with distilled water as the solvent [25,26,27]. MLE can also be obtained from an ethanolic solvent [28,29], but the aqueous MLE is safe, cheap, easy to prepare, and has been applied by farmers.

The aqueous MLE is composed of phytohormones, mineral nutrients, proteins, vitamins, phenolics, and others [30,31]. El Sheikha et al. [31] recorded the presence of gibberellin, auxin, and cytokinin in MLE. Additionally, several essential and non-essential nutrients were found in MLE, including nitrogen (N), phosphorus (P), potassium (K), calcium (Ca), magnesium (Mg), copper (Cu), iron (Fe), zinc (Zn), manganese (Mn), selenium (Se), and sulfur (S) [32]. Arif et al. [33] noted that MLE is rich in ascorbate and amino acids. Regarding bioactive compounds, the aqueous and ethanolic extract of moringa leaf were reported to contain phenolic, flavonoid, and saponin compounds [34]. 

Furthermore, MLE can be applied as a seed treatment, foliar application, and root application [32], but the foliar application is the most prevalent method. It is recommended to apply the biostimulant via foliar spraying in the early morning or late afternoon when the stomata in the leaves are open [35].

## 3. Mechanism of MLE in Improving Quality

**Figure 1:**
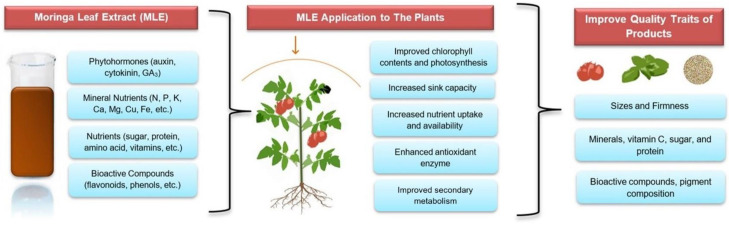
Mechanism of moringa leaf extract (MLE) in improving quality traits.

Figure 1 presents several mechanisms of MLE in improving product quality. According to Colla et al. [36], biostimulants contain beneficial components that have positive effects on nutrition, photosynthesis, and secondary metabolism in plants, improving product quality. Since MLE has considerable amounts of minerals, it promotes nutrient uptake and improves the nutrient status and product quality of the plant [37]. As expected, the MLE application also provides higher photosynthetic pigment and a photosynthesis rate that contributes to the enhancement [38,39]. The positive photosynthesis results might also be related to leaf area increment promoted by the MLE application since it occurs in the leaf [18]. The biostimulant may activate the signaling pathway, which modulates the expression of gene-encoding enzymes associated with secondary metabolism [40]. This is in line with research by Nasir et al. [41], who confirmed that MLE is rich in vitamin C, and when applied exogenously, it enhances the endogenous vitamin C of fruit. These results imply that MLE alters plant metabolism by increasing vitamin C synthesis in the leaf and transporting it to the fruit through the phloem [41,42]. In addition, the amino acids present in biostimulants also play vital roles in increasing the direction and rate of metabolic processes [43].

The presence of phytohormones in a biostimulant influences the physiological process in a plant [3]. MLE possesses phytohormones, such as auxin and GA_3_, with special attention to cytokinin [44]. Cytokinin plays a vital role in increasing sink capacity [45]. This is in accord with earlier observations in which MLE has been demonstrated to stimulate the translocation of assimilates from the leaf to the parts that modulate quantity and quality [39]. 

Enhancing stress tolerance in plants helps to maintain the quality of the harvested product under stress conditions [46]. MLE is an important source of natural antioxidants that positively improves antioxidant compounds in the product [47] and affects the plant defense system against oxidative stress [48]. This is because it has specific antioxidant mechanisms in terms of stress tolerance [49]. The ability of MLE to alleviate oxidative stress was found in relation to preserving the quality of fresh products and cut flowers. The application of MLE under stressed conditions induced antioxidant enzyme activities that promoted the quality attributes of fresh products in several crops [50,51]. In cut flowers, it enhances the activities of antioxidant enzymes (CAT and POX), which play marked roles against oxidative stress, inhibiting the floret senescence [52]. Besides antioxidant enzymes, delayed flower senescence was linked to the effectiveness of MLE in decreasing MDA content, an indication of reducing lipid peroxidation and maintaining membrane stability [53]. 

## 4. Effects of MLE on Physical and Sensory Quality 

The physical properties of agricultural products, such as size, color, firmness, and shape have been used as the criteria for the initial evaluation during sorting and grading. Moreover, sensory attributes are also important for consumer satisfaction. Sensory traits can include texture, appearance, taste, or aroma.

### 4.1. Weight and Size

Recent studies reported that MLE positively influenced fruit weight and size. Results of a study on a grapevine treated with 2.5% and 3.5% MLE in vegetative growth provided a higher berry weight and diameter [21]. Furthermore, foliar spraying of 3% MLE at different growth stages enhanced the size of ‘Kinnow’ mandarin fruit, likely due to the presence of zeatin, the most common cytokinin in MLE [41]. Zeatin accelerates cell division and cell enlargement during fruit development [54]. Cytokinin also improves sink capacity and photosynthate assimilation as a result of more extended green areas in the leaf [45]. A higher fruit weight and size might also be associated with the role of minerals in MLE, such as K and Zn. K regulates the accumulation of starch and sugar, while Zn, a tryptophan precursor, is involved in IAA synthesis, which is required for the growth and development of fruit [55].

In tomatoes, foliar spraying with MLE (25 mL per plant) at two-week intervals yielded maximum nutrient uptake and, consequently, led to heavier fruit [56]. Similar effects were observed in brinjal fruit [37]. Hala and Nabila [39] evaluated the application of MLE in sweet pepper. Plants treated with MLE at different concentrations provided higher fruit lengths, diameters, and weights. 

Additionally, the effects of MLE on the physical qualities of ornamental plants have been documented. Foliar spraying of MLE and corm soaking increased the inflorescence quality of *Freesia hybrida* [57]. Likewise, the maximum flower diameter of *Helianthus annuus* was noted in plants treated with 50% MLE [58]. It was found that cytokinin plays a major role in flower development and quality [59].

### 4.2. Firmness

Firmness is one of the most important parameters for the shelf-life and market value of the product. Foliar spraying of 3.5% MLE increased berry firmness due to the beneficial constituents in MLE, such as macronutrients, micronutrients, and cytokinin [21]. A similar conclusion was found by Abo El-Enien et al. [60], i.e., foliar spraying of MLE significantly enhanced fruit firmness of the navel orange. The application of 10% MLE together with 2% B and 3% Ca improved the firmness of plum fruit [61]. Moreover, Ismail and Ganzour [22] reported an increment in fruit firmness of strawberries after spraying solely with MLE or in combination with KNO_3_. They stated that Ca is involved in cell wall formation and plays a binding role in the complex polysaccharides as well as proteins. Martins et al. [62] also mentioned the role of Ca in the stability of cell walls. Similarly, Thanaa et al. [63] showed that the increase in fruit firmness of plums might be a consequence of the high Ca level in MLE.

### 4.3. Color and Sensory Attributes

Color is a visual feature that proves the product quality. So far, little is known about the effect of MLE on the color parameters of a product. According to Thanaa et al. [63], foliar spraying with 6% MLE resulted in higher lightness (L*) and hue angle (h°) value of plum fruit, which was likely due to the increased enzyme activity and anthocyanin content by the MLE application, leading to the formation of more colored fruit. These results are in agreement with the findings by Mahmoud et al. [61], who found an increase of L* in Hollywood plum cultivar. 

In addition, studies that investigate the effects of MLE on sensory attributes are rare. Khan et al. [64] demonstrated the foliar application of 3% MLE on five grapevine cultivars. The treatment did not influence consumer acceptance of the flavor or color of the berries; rather, it improved the taste, texture, and overall acceptability in all tested cultivars. This study also revealed that phenolic compounds may regulate the sensory traits of fruit. 

## 5. Effects of MLE on Nutritional Quality 

Nutritional value is a highly important quality trait that cannot be tasted, seen, or felt. It includes carbohydrates, protein, fat, minerals, and vitamins that are essential to human health.

### 5.1. Carbohydrates

Carbohydrates are composed of simple sugars and are usually stored as starches in plants. The improvement in the starch content of maize grains by foliar application of MLE was reported by Chattha et al. [65] and Kamran et al. [66]. Foliar application of MLE was quite effective at increasing the starch content of maize grains harvested from plants grown under chilling stress [67]. Moreover, MLE treatment at several concentrations produced higher carbohydrates in flax seeds grown under newly reclaimed sandy soil, thanks to the presence of cytokinin in the MLE [68]. 

Furthermore, sugar directly affects the sweetness of fruits and vegetables. In citrus, foliar spraying of 3% MLE alone or in combination with 0.25% K_2_SO_4_ + 0.6% ZnSO_4_ intensified the total sugar of the fruit, due to the existence of zeatin in MLE that stimulates sugar translocation to the fruit [55]. MLE possesses considerable amounts of starch, sugar, zeatin (involved in the relation of source–sink), and minerals that contribute to improving sugar accumulation in fruit [41]. Adding MLE alone or in combination with glycine and folic acid enhanced the sugar content of grape cv. Flame Seedless [69]. Furthermore, MLE application at bloom, in addition to the fruit set and premature stages, positively influenced total sugar, as well as the reducing and non-reducing sugar content in grape berries [64]. An increase in sugar content of snap bean pods was observed in the MLE-treated plant due to the increment in photosynthetic pigment concentration [70].

### 5.2. Protein and Fat

Basra and Lovatt [71] reported that fruit from the tomato plant treated with foliar and root application of MLE had greater soluble protein concentration compared to the control. Ashraf et al. [72] applied a foliar application of 3% MLE continually for 15-day intervals and found an increase in the protein content of radish. Consistent results were obtained in peas [73], quinoa [74], and wheat [75]. Moreover, MLE application alone or in combination with K provided greater protein content in chickpea grains [23]. Under heat stress conditions, wheat cv. Fsd produced higher grain protein with a 3% MLE application compared to other biostimulants [76]. Furthermore, the maximum total soluble protein in the spinach leaf was observed after the foliar application of MLE and might be linked to several vitamins, minerals, and cytokinin content in MLE [47]. Protein content in maize grain was higher in response to the foliar application of 3% MLE at knee height, tasseling, and the grain-filling stage compared to the control [77]. Similarly, MLE improved the grain protein of maize, while the maximum value was obtained when it was combined with sorghum water extract [66].

However, there is a lack of studies about the effects of MLE on the fat composition of a product. A study by El Sheikha et al. [31] revealed that MLE treatment improved linoleic acid content in snap bean pods, which is one of the omega fatty acids that prevents obesity, reduces cholesterol, and enhances the immune system.

### 5.3. Vitamin C

Recent studies confirmed the beneficial effects of MLE application on the vitamin C content of products. In citrus fruit, MLE application—before flowering and at fruit set stages—exhibited maximum vitamin C content, about 1.16-fold higher than the control [41]. MLE application also significantly increased vitamin C in grape berries [64]. Foliar spraying of MLE showed higher vitamin C in broccoli inflorescence under normal and water stress conditions [78]. In addition, the use of 6% MLE successfully increased the vitamin C content in lettuce cv. Great Lakes that were grown under a glasshouse [79]. Considering that MLE is rich in vitamin C, its application via foliar spraying enhanced the endogenous vitamin C of cucumber fruit [80]. Similarly, an increment of vitamin C content in strawberries was noted in MLE treatment as a result of high protein content in MLE that facilitated ascorbic acid formation [22].

### 5.4. Mineral Nutrients

Minerals are usually distinguished as macro- and micronutrients. Macronutrients include nitrogen (N), phosphorus (P), potassium (K), calcium (Ca), and magnesium (Mg). Meanwhile, micronutrients consist of manganese (Mn), iron (Fe), copper (Cu), zinc (Zn), sodium (Na), cobalt (Co), chlorine (Cl), fluorine (F), iodine (I), sulfur (S), and selenium (Se).

According to a previous study, MLE-treated plants exhibited higher N, P, and K content in a snap bean pod [70]. Similarly, foliar application of MLE markedly improved N, P, K, and S content in brinjal fruit [37]. This treatment also produced the highest K and C levels in sweet pepper fruit [39]. Alkuwayti et al. [81] examined the mineral content in a basil leaf treated with MLE. A plant treated with 5 g L^−1^ via a foliar application of MLE yielded higher N, P, and K content in the first cutting. The same trend was observed in pea seeds, where MLE treatment displayed higher nutrient accumulation, including N, P, K, Ca, Mg, and Fe [73]. According to Abdalla [82], MLE accelerates nutrient uptake by improving root membrane permeability and increasing nutrient mobility. 

Several studies also showed the beneficial effects of MLE treatment on mineral nutrients in the stevia leaf. Sardar et al. [30] noted that foliar application of 20% MLE improved the leaf’s N, P, K, Ca, Mg, Na, and Zn accumulation. Furthermore, Jain et al. [83] applied leaf extract from different moringa varieties via foliar spraying and observed an increase in the leaf’s N, K, and Ca concentrations under greenhouse conditions. 

Recent studies were conducted to verify the mineral improvement in quinoa seeds via MLE application. Rashid et al. [74] and Rashid et al. [84] observed increments in the Ca and K of the seeds using the foliar application of MLE. These results might be associated with the fact that MLE improves the diversion of assimilates from the leaf to the seed and is an excellent source of minerals, antioxidants, and secondary metabolites; hence, it has the ability to maintain mineral content in plant tissue. Likewise, regarding P, S, Zn, and Fe levels in quinoa seeds, the highest values of these traits were noted in plants treated with 3% MLE at the anthesis stage [85]. Nevertheless, no significant enhancement for seed mineral content was found by the foliar spraying of stored MLE, except for Mn [86].

### 5.5. Nitrate

Nitrate is naturally present in vegetables and fruits. It has beneficial health effects considering its role in vascular and immune function [87]. However, green leafy vegetables generally contain high nitrate levels [88]. The presence of high nitrate levels, above a certain threshold, is considered harmful to human health [89]. Previous studies revealed that MLE treatment reduced nitrate content in several horticultural commodities although its mechanism of action is still unknown. The foliar application of 3% MLE decreased nitrate and nitrite content in navel orange fruits [60]. In similar reports, Yaseen and Takácsné Hájos [90] showed that MLE reduced nitrate in lettuce. Toscano et al. [91] demonstrated the effects of MLE on nitrate content in the baby leaves of kale and broccoli. In these cases, MLE significantly reduced nitrate content (−70%) in kale, while it increased at about 60% in broccoli. However, it remained under the maximum permitted threshold of the European Commission Regulation. 

## 6. Effects of MLE on Chemical Quality 

The chemical qualities of agricultural products are related to total soluble solid, titratable acidity, bioactive compounds, and pigment compositions.

### 6.1. Total Soluble Solid and Titratable Acidity

Total soluble solid (TSS) and titratable acidity (TA) are two components that determine the commercial value of a product. A study conducted on citrus, pears, and grapes demonstrated the effects of MLE on TSS and TA. The enhancements in the TSS and TA of citrus were attained on a plant treated with 3% MLE, as MLE is rich in starch and sugar [55]. A similar trend was obtained by Abo El-Enien et al. [60] on navel orange fruit. Moreover, when pear trees were treated with MLE, they had higher fruit TSS and reduced acidity [92]. In contrast, MLE at a concentration of 2.5% did not significantly impact the TSS of the grape berry [21]. These results are in agreement with research by Nasir et al. [41], who reported that TSS in citrus fruit was not influenced by MLE application. They confirmed that this treatment produced larger fruit sizes, while higher TSS was often found in smaller fruits and vice versa.

### 6.2. Bioactive Compounds

According to recent findings, the application of MLE was effective at increasing the total phenolic content of broccoli [78] and radishes [72]. Under a glasshouse experiment, increments in polyphenols of different lettuce cultivars were found in plants treated with 6% MLE [79]. MLE application at a concentration of 20% displayed the maximum phenolic and flavonoid content in the stevia leaf [30]. Furthermore, Aslam et al. [47] evaluated several bioactive compounds in spinach after MLE application. The greatest results on total phenolic and phenolic acid content were obtained on MLE treatment due to the presence of flavonoid, quercetin, and kaempferol in MLE. In addition, the application of MLE containing minerals and vitamins influenced plant metabolic processes and, consequently, increased total phenolic content [24]. In a study with fennel fruit, foliar spraying with MLE significantly enhanced total phenolic content because phenolic compounds in MLE influenced the endogenous phenol content in fruit [27]. Additionally, radish seeds previously hydro-primed with 5% MLE for 5 h showed the highest polyphenol content in the root [93].

Apart from increasing flavonoid and phenolic content, MLE has been reported to alter other bioactive compounds in products. The application of 5 g L^−1^ MLE increased the medicinal value of the basil leaf, which was expressed by higher major bioactive compound percentages, including eucalyptol, linalool, estragole, and caryophyllene [81]. In basil, estragole and linalool have anti-inflammatory, antimicrobial, and antidematogenic properties [94,95]. The beneficial effects of foliar spraying with aqueous MLE were also reported on the essential oils of fennel, such as myrcene and 1.8 cineol [29]. Similarly, the treatment of MLE appreciably enhanced volatile oil compounds of geranium, for example, linalool, citronellol, and geraniol [51]. 

### 6.3. Pigment Composition

The pigment compositions of agricultural products produce specific colorations, which are visual quality traits. In addition, they have roles in human health. For example, anthocyanin is involved in the prevention of cardiovascular and neurodegenerative diseases [96]. Similarly, chlorophyll and carotenoid have important roles in preventing different diseases, such as cardiovascular diseases, cancer, and other chronic diseases [97].

Previous studies demonstrated the beneficial effects of MLE application on the pigment compositions of several horticultural products. Alkuwayti et al. [81] found that the increments of MLE concentrations were followed by increases in chlorophyll content. This may be linked to the role of Mg in the extract as a chlorophyll constituent. Furthermore, MLE improved the carotenoid and chlorophyll content of spinach due to the hormone, mineral, and vitamin roles in MLE, which delay leaf senescence [47]. Biostimulants, including MLE, can also delay flower senescence and maintain the pigment composition of cut flowers [53,98]. Toscano et al. [91] confirmed that the improvement in chlorophyll and carotenoid contents were closely associated with the high amount of carotenoids (xanthin, α-carotene, ß-carotene, and lutein) and chlorophyll in MLE. Recent reports revealed an increase in anthocyanin content in grape berries [69], quinoa seeds [85], and roselle calyx [99]. Moreover, total lycopene was significantly greater in tomato fruit harvested from a MLE-treated plant [71].

## 7. Conclusions and Future Prospective

Based on the above findings, it is well documented that MLE is a good source of phytohormones, minerals, secondary metabolites, and bioactive compounds. These beneficial components alter the physiological processes in plants, such as increasing photosynthesis, nutrient uptake, sink capacity, antioxidative enzymes, and secondary metabolism in plants, contributing to the quality improvements of agricultural products (Table 1). Additionally, MLE is environmentally friendly and inexpensive. Nevertheless, studies about the effects of MLE on quality attributes have been dominated by foliar application methods. Further studies are needed to explore other MLE application methods, such as seed treatments or soil applications. Therefore, research and development should continue to extend the potential utility of MLE as a biostimulant.

## Figures and Tables

**Table 1:** Overview of several quality traits of different plants in responses to moringa leaf extract (MLE) application.

Plants	Parts	Methods	Concentration	Responses	References
Radish	Root	Foliar spray	3 and 5%	Increased protein, crude fiber, and ash content.	[72]
Quinoa	Seed	Foliar spray	3%	Increased Ca, K, and protein.	[84]
Flax	Seed	Foliar spray	10, 20, and 30%	Increased carbohydrate content.	[68]
Maize	Seed	Foliar spray	3%	Increased protein and starch.	[66]
Pea	Seed	Foliar spray	1, 2, 3, 4%	Enhanced protein, N, P, K, Ca, Mg, and Fe contents.	[73]
Lettuce	Leaf	Foliar spray	6%	Reduced nitrate and improved polyphenol content.	[79]
Basil	Leaf	Foliar spray	5 g L^−1^	Improved N, P, K concentrations; bioactive compounds (eucalyptol, linalool, estragole, caryophyllene), chlorophyll, and carotenoids.	[81]
Spinach	Leaf	Foliar spray	1:30	Enhanced total soluble proteins, chlorophyll, carotenoids, total phenolic content, and total phenolic acid.	[47]
Stevia	Leaf	Foliar spray	10, 20, and 30%	Improved N, P, K, Ca, Na, Mg, Zn, Fe, phenol, chlorophyll, carotenoid, and flavonoid content.	[30]
Baby leaf of kale	Leaf	Foliar spray	200 mg L^−1^	Decreased nitrate and improved total polyphenol as well as sugar.	[91]
Rocket	Leaf	Foliar spray	1, 2, 3%	Enhanced chlorophyll, carotenoid, protein, sugar, phenol, and ascorbic acid content.	[82]
Broccoli	Flower	Foliar spray	200 mg L^−1^	Improved diameter, weight, carbohydrate, ascorbic acid, and phenols.	[78]
*Freesia hybrida*	Flower	Foliar spray Corm soak	1, 2, and 3% 1, 2, 5, and 10%	Increased flower diameter and quality of inflorescences. Increased quality of inflorescences.	[57]
Sunflower	Flower	Foliar spray	25 and 50%	Improved flower diameter.	[58]
Gladiolus	Cut flower	As holding solutions	1, 2, 3, and 4%	Maintained chlorophyll as well as relative water content, suppressed microbial growth, and improved vase life.	[52]
Rose	Cut flower	Pulsing treatment	1:10, 1:20, 1:30, and 1:40	Maintained the relative water content, suppressed microbial growth, and enhanced total phenol and vase life.	[53]
Snap Bean	Pod	Foliar spray	1:30	Increased length, diameter, weight, protein, linoleic acid, sugar, and several amino acids, N, K, Mg, S, P.	[31]
Citrus	Fruit	Foliar spray	3%	Increased weight, size, sugar, TSS:TA ratio, ascorbic acid, and phenolic content.	[41]
Strawberry	Fruit	Foliar spray	2, 4, and 6%	Improved weight, firmness, TSS, TSS:TA ratio, and anthocyanin content.	[22]
Plum	Fruit	Foliar spray	4, 5, and 6%	Enhanced weight, length, firmness, and color attributes.	[63]
Brinjal	Fruit	Foliar spray	1:30	Increased weight, length, and N, P, K, S content.	[37]
Grapevine	Fruit	Foliar spray	2.5 and 3%	Improved firmness, diameter, and titratable acidity.	[21]
Tomato	Fruit	Foliar spray and root application	3.3%	Improved total sugar, protein, and lycopene.	[71]
Sweet pepper	Fruit	Foliar spray	2, 4, and 6%	Enhanced fruit length, diameter, total vitamin C, carbohydrate, K, and Ca content.	[39]

## Data Availability

Not applicable.
